# Characteristics of Doped TiO_2_ Nanoparticle Photocatalysts Prepared by the Rotten Egg White

**DOI:** 10.3390/ma15124231

**Published:** 2022-06-15

**Authors:** Chung-Ming Lu, Raju Kumar Sharma, Pin-Yun Lin, Yi-Hsun Huang, Jung-Sheng Chen, Wen-Chien Lee, Chien-Yen Chen

**Affiliations:** 1 Department of Chemical Engineering, National Chung Cheng University, 168 University Road, Min-Hsiung, Chiayi 62102, Taiwan; n0935718023@gmail.com; 2 Department of Earth and Environmental Sciences, National Chung Cheng University, 168 University Road, Min-Hsiung, Chiayi 62102, Taiwan; raju28212@gmail.com (R.K.S.); x699237014x@gmail.com (Y.-H.H.); 3 Department of Chemistry and Biochemistry, National Chung Cheng University, 168 University Road, Min-Hsiung, Chiayi 62102, Taiwan; kittycat1604@hotmail.com; 4 Department of Medical Research, E-Da Hospital, Kaohsiung 82445, Taiwan; ed113187@edah.org.tw; 5 Center for Innovative Research on Aging Society, AIM-HI, National Chung Cheng University, 168, University Rd., Min-Hsiung, Chiayi 62102, Taiwan

**Keywords:** TiO_2_ nano-powders, F-doped TiO_2_ nanoparticles, sol–gel method, photocatalyst

## Abstract

In this study, expired egg white was used as a template, and a sol–gel method was employed to prepare pure-phase TiO_2_ nano-powder and mixed-phase powders doped with NaF and NaI. The influences of different calcination temperatures, doping elements, and doping amounts during the preparation process on the photocatalytic performance and activity of the prepared TiO_2_ powders were studied. The results of the experiments showed that the F-doped TiO_2_ had the highest photocatalytic activity when the doping amount was 1.2%, as examined by EDS, where the sintering temperature was 500 °C. F-doped TiO_2_ nanoparticles were also synthesized by the sol–gel method using tetrabutyl titanate and NaF mixed with expired egg white protein as the precursor. The F-TiO_2_ photocatalyst was characterized using FE-SEM, HR-TEM, EDS, XPS, and UV-Vis, and the photocatalytic activity was evaluated by photodegradation of methylene blue under visible light. The results showed that doping with F reduced the energy band gap (3.04 eV) of TiO_2_, thereby increasing the photocatalytic activity in the visible-light region. The visible-light wavelength range and photocatalytic activity of the catalyst were also affected by the doping amount.

## 1. Introduction

In 1972, Fujishima and Honda discovered that irradiated TiO_2_ particles can cause continuous oxidation–reduction reactions in water. Taking this as an opportunity, a new era of heterogeneous catalysis research began. Since the 1990s, great progress has been made in TiO_2_ photocatalysis research in terms of the photocatalytic removal of water- and gas-phase organic and inorganic pollutants in the field of environmental protection, and this is considered to be a promising technology for deep purification of environmental pollution. In particular, the recent COVID-19 virus is raging, and there have been related studies using TiO_2_ photocatalysis that have made research contributions in the field of health and hygiene [1].

Due to the wide band gap of TiO_2_, the catalytic activity is generally not high under sunlight. The material needs to be scientifically modified so that the material can produce catalysis in the range of visible light. For this reason, many researchers have assessed various scientific methods for the preparation and modification of TiO_2_ photocatalysts, using different preparation methods or preparation conditions, in addition to related technologies such as doping of different elements, semiconductor compounding, and the introduction of template agents. Through this research, the photocatalytic activity of TiO_2_ may be able to be improved, and semiconductor-based research on photocatalysis has received extensive attention [2,3,4,5,6,7]. Element-doped TiO_2_ is regarded as a promising way in which to obtain a more effective photocatalyst. In this study, F-doped TiO_2_ was synthesized by the sol–gel method, demonstrating both a high adsorption capacity and high photocatalytic activity under simulated sunlight irradiation. Most prior studies have been carried out under ultraviolet (UV) light, because TiO_2_ photocatalysts show a relatively high activity and chemical stability under UV light, exceeding the band gap energy in the rutile or anatase phase of 3.0 or 3.2 eV, respectively. As this part of UV light (300~400 nm) only accounts for 4~6% of solar energy, the utilization rate of solar energy is quite low. Considering practical application, the visible light activity of TiO_2_ must be improved. For this reason, related research has used metal or non-metal doping to modify the properties of TiO_2_ in order to improve the visible light utilization of TiO_2_. Since Asahi, et al. [8] reported that non-metallic N-doped TiO_2_ achieved visible light activity, research into non-metal doping has been very active [9,10,11], in particular, the use of expired eggs as a template [12] in doped TiO_2_ research has also been reported.

Sol–gel method refers to the precursor substances such as water-soluble salt or oil-soluble alkoxide dissolved in water or organic solvent to form a homogeneous solution. Interestingly, the nanomaterials can be prepared as smaller and more uniform particles at room temperature, which meets the conditions of simple industrial manufacturing and lower cost. Due to the low temperature of this preparation method, large doses of inorganic and organic substances are allowed to be doped [13,14,15,16]. However, a new type of element-doped TiO_2_ photocatalyst was prepared using sol–gel method, and this study also analyzed the mechanism of TiO_2_ photocatalysis, examined related preparation methods, and investigated methods by which to improve its photocatalytic activity, such as doping and compounding, with the aim of further expanding the application of TiO_2_ photocatalysis in the future. The sol–gel preparation method used in this work is popular because it is simple, less time-consuming than other methods, and operates under normal atmospheric temperature, and the process of synthesizing nanomaterials is easy and convenient [6,17,18]. The new F-doped TiO_2_ photocatalytic catalyst prepared in this work was tested under UV-visible irradiation and in MB photo reactivity experiments, and it was found to have a good reaction effect in the visible light range. At the same time, we used various analytical instruments, including FE-SEM, HR-TEM, XPS, and EDS, and compared the doped TiO_2_ photocatalyst with commercial-grade P-25 TiO_2_ powder to verify the relationship between its chemical properties and physical properties. In addition, looking at recent research reports, F-doped TiO_2_ material has a high “energy-saving” value. In order to reduce the energy consumption required for cooling in the hot summer, it would be of great value to find a new type of building near-infrared shielding material. F-doped TiO_2_ nanocrystals represent a new type of energy-saving window coating with an enhanced near-infrared (NIR) shielding performance [19]. In addition, F-doped TiO_2_ also has a high “environmental protection” value. The degradation of toxic dyes by F-doped TiO_2_ can be improved by photochemical activity [20].

With an eye to attaining the objective of a green approach in nanobiotechnology, an expired egg white protein (EWP) biological template was used as the raw material from which to derive the precursor solution for synthesis of the photocatalyst nanoparticle material. A simple sol–gel process was used in a bottom-up approach, which involved testing different solution pH values, solution concentrations, drying times, and calcination temperatures for the synthesis of TiO_2_ nanomaterials. The physicochemical properties of the various photocatalyst TiO_2_ synthetic particles obtained were studied, such as the crystal structure, doping of different substances, absorption rates in the UV and visible light ranges, and under simulated sunlight ranges and their morphology, such as the shape and size of the catalyst particles, etc.

## 2. Materials and Methods

### 2.1. Chemicals and Reagents

Chemical reagents NaF (sodium fluoride, 99% purity; Acros, Geel, Belgium) and NaI (sodium iodide, 99% purity; Alfa Aesar, Ward Hill, MA, USA) were used to prepare the doping substances for use with the precursor solution. 25 wt.% of AA, ethyl alcohol (99.5%; Assay, Tainan city, Taiwan) and titanium (IV) n-butoxide, 99.0% purity; Acros) were used for material synthesis. Methylene blue (96.0% purity; Fluka, Dresden, Germany) was used to prepare a 10ppm blue aqueous solution for photocatalytic reaction testing. All chemical reagents were used without further purification.

### 2.2. Preparation of TiO_2_ Nanoparticles Using Egg White Protein

Using titanium (IV) n-butoxide as the main raw material, absolute ethanol as the dispersion medium, and acetic acid as the inhibitor, gels of titanium dioxide and doped TiO_2_ were prepared under different reaction conditions. The absolute ethanol and butyl titanate were mixed thoroughly and uniformly to prepare solution A. To prepare solution B, absolute ethanol, acetic acid, and expired egg white (or deionized water) were mixed evenly. Then, under vigorous stirring, solution A was added to solution B slowly in a dropwise manner and stirred for 30 min more. After the addition was completed, stirring was further continued approximately 5 min to form the gel, followed by aging for several hours at room temperature to obtain a wet gel of titanium dioxide. This wet gel was then placed in a drying box for several hours to remove the water, organic solvents, and reaction by-products in the gel to obtain TiO_2_ as a dry gel. The dry gel of TiO_2_ was fully grounded and calcinated at 500 °C using muffle furnace. In the heating process, the dry gel product first removes the water and alcohol adsorbed on the surface at low temperature, and then oxidizes the alkoxy groups at 280~300 °C. Furthermore, at a temperature above 400~500 °C (after more than 5 h), the hydroxyl group in the sample structure is removed. Via the calcination process, an active TiO_2_ catalyst was formed [21].

### 2.3. Doping of NaF and NaI in TiO_2_ Powder

Solution A and solution B were prepared separately, as described above. In case of NaF and NaI doping, the prepared solution B was doped with 0.5 g of NaF/NaI in 20 mL of double deionized water. To be continued, the prepared solution was uniformly mixed with above prepared solution A at the same time, using ultrasonication to form the dry gel. The formation of dry gel was dried to remove the water and it becomes a dry solid block. It should be noted that the drying time required after the sol state has formed varies according to the type of doped TiO_2_ catalyst to be prepared. For example, when using EWP as the template for the F-doped TiO_2_ catalyst, a longer drying time is required, because when acetylacetone is added, the proper precursor is obtained by controlling the rate of the hydrolysis reaction of Ti(OBu)_4_, and the drying time required for this production process is 2~3 days. We carefully mashed the solid block material with an agate mortar, grinding it into as fine a powder as possible, and calcinated at 500 °C. In the process of heating, drying, and calcining the samples containing doping-compound and EWP, the dried gel material first removes the water and alcohol adsorbed on the surface at a low temperature, and then oxidation of the alkoxy group occurs at about 300 °C, and then at a higher temperature of about 500 °C (after 5 h or more), the hydroxyl group in the structure is removed. Therefore, in the process of calcination, an active doped TiO_2_ catalyst is formed, such as the successful F-doping of TiO_2_.

### 2.4. Photocatalytic Degradation of Methylene Blue on Photoirradiation of TiO_2_ Powder

In order to confirm the degradation ability of the prepared TiO_2_ photocatalyst, a dye photocatalytic degradation experiment was carried out. First, a total of 500 mL of 10 mg/L methylene blue solution was prepared and placed in five 50 mL light bottles. Then, 40 mL of MB were added to each of the five bottles, which contained 40 mg of catalyst, and the bottles were subjected to magnetic stirring. After standing for 10/30 min in a dark room, the photocatalytic status of the samples was examined when not illuminated.

The photocatalysis test was then performed again, using a light source consisting of a kind of simulated sunlight through a filter. The reaction time under the laboratory-simulated visible light was observed after ten minutes, where 1 mL of samples were taken every ten minutes, and each removed sample was subjected to high-speed centrifugation at a rotating speed 5000× *g* rpm for 5 min to obtain the supernatant, and a UV-Visible spectrophotometer with a wavelength of 665 nm was employed for analysis of each supernatant samples.

### 2.5. Characterization

#### 2.5.1. Analysis of Crystallization, Particle Size, Appearance, and Surface Dispersion of the Synthesized Catalyst by Scanning Electron Microscopy (SEM)

The doping component within the TiO_2_ structure, particle shape, and surface distribution of the synthesized materials was observed and determined by SEM following the procedure of Bayan et al. [22]. The surface morphology of the TiO_2_ photocatalyst was evaluated using a field emission scanning electron microscope (FE-SEM; Hitachi S4800-I instrument, Tokyo, Japan) with a coating of precious metal Pt at the operating range of 0.1–30 kV [23].

#### 2.5.2. Estimation of Morphology of Synthesized Material by HR-TEM-EDX

Transmission electron microscopy (TEM) was used to observe the particle size of the catalyst and the distribution of the doped substances, and selected area electron diffraction (SAED) was employed to analyze whether the material was polycrystalline, single-crystal, or two-crystal mixed-phase. The instrument was equipped with an X-ray energy dispersive spectrometer (EDS), which was employed for elemental qualitative and quantitative analysis. Morphological study of the synthesized TiO_2_ photocatalyst was conducted using a high-resolution transmission electron microscope (HR-TEM) (JEOL JEM-2010, Chiayi, Taiwan) with an acceleration voltage of 200 kV [24].

#### 2.5.3. Estimation of Elemental Quantification of Synthesized Material by XPS

The chemical state of the atoms on the surface of the photocatalyst and elemental quantification of the synthesized material were estimated by high-resolution X-ray photoelectron spectroscopy (XPS; PHI Hybrid Quantera, Chanhassen, MN, USA), which analyzes the elements and chemical bonds on the surface of a material and is used to perform elemental quantification. Al Kα radiation was used in XPS as a monochromatic X-ray beam of light with 49.3 W, 45.0°, and 280.00 eV.

#### 2.5.4. Estimation of Synthesized Material Absorption by UV-VIS-NIR

UV/visible spectrometry (UV-VIS-NIR) analyzes the absorbance of light, and the results were helpful to inform the selection of the light source in this study. This instrument uses BaSO_4_ as the background value to zero, and the catalyst powder is then placed in the middle of the instrument. When UV and visible light irradiate the surface of the catalyst separately, electrons in different molecules absorb different energies and produce refraction, reducing the refractive index and wavelength. The results were drawn into a graph, and the refractive index graph was converted into a yield graph through the Kubelka–Munk formula. The absorption of TiO_2_ and the doped TiO_2_ synthesized material was estimated using UV-VIS-NIR. A UV-VIS-NIR spectrometer (HITACHI U4100, Japan) was used to record (wavelength range = 200–2600 nm) the reflectance spectra of the samples, with BaSO_4_ as a reference sample and with constant temperature through cooling Pbs Gain detector.

#### 2.5.5. Photocatalytic Activity Measurements

A photocatalytic reactor was equipped with a simulated sunlight lamp, and the photocatalytic activity of the catalyst was characterized by the degradation rate of methylene blue solution under irradiation by the 60 W lamp. Experiments were carried out at room temperature in quartz glass beakers. Forty ml of MB aqueous solution was added to glass bottles containing 40 mg of TiO_2_ catalyst, and the bottles were subjected to magnetic stirring. After irradiation for a period of time under a simulated sun lamp at a distance of 10 cm, the samples were centrifuged, and the supernatants were removed. The absorbance was measured using a PRO739, TU-1900 dual-beam UV-visible spectrophotometer. Using the absorbance and concentration curves of methylene blue, the concentration of the methylene blue solution after degradation was obtained.

## 3. Results and Discussion

### 3.1. Morphologic Analysis of Material by SEM

#### 3.1.1. Effects of 400 °C and 500 °C Calcination

**Figure 1:**
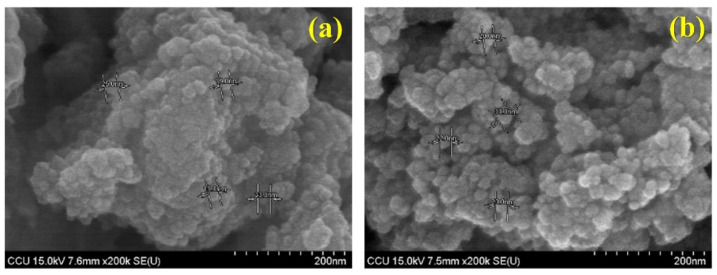
Field emission-scanning electron micrographs of bio-templated TiO_2_ nanoparticles: (**a**) 400 °C and (**b**) 500 °C.

Figure 1 below presents SEM images of TiO_2_ photocatalysts prepared at different calcination temperatures (400 °C and 500 °C). It can be seen from the figure that the TiO_2_ prepared at the higher calcination temperature presented a relatively uniform granular morphology and had a good dispersibility and a small agglomerate size of only 40–100 nm. The TiO_2_ prepared by calcination at the lower temperature had a poor dispersibility, and there was more obvious agglomeration, with an agglomerate size of 100–300 nm. Therefore, according to the experimental results, when comparing the material after calcination at 400 °C and 500 °C, as the calcination temperature increased, the degree of agglomeration of TiO_2_ decreased.

#### 3.1.2. Analysis of TiO_2_ and F-Doped TiO_2_ by SEM

**Figure 2:**
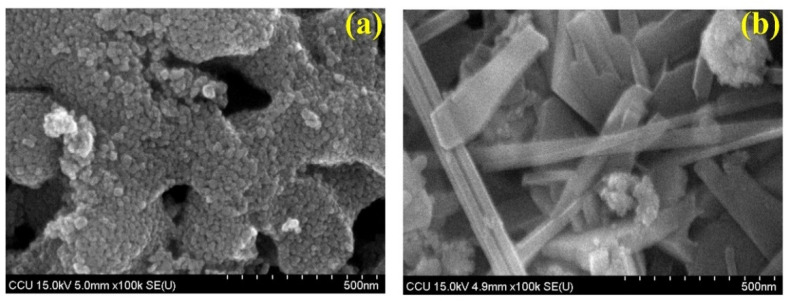
Field emission-scanning electron micrographs of bio-templated photocatalyst at 500 °C: (**a**) TiO_2_ and (**b**) F-doped TiO_2_.

Figure 2 below presents SEM images of the TiO_2_ and F-doped TiO_2_ photocatalysts. It can be seen from the figure that the TiO_2_ modified by F-doping had a uniform granular morphology, with good dispersibility and a small agglomerate size. After calcination at 500 °C, the agglomerates were observed to be only 10–30 nm in size. However, the unmodified TiO_2_ had a poor dispersibility, and there was more obvious agglomeration after calcination at 500 °C, the size of the agglomerates being 30–50 nm. More notably, the F-doped TiO_2_ crystal shape created in our laboratory is not mentioned in the current literature, and this can therefore be said to be a pioneering work. The shape obtained was particularly strange; however, photo-reactivity testing using MB strongly proved the effects to be the same. According to the results of the experiment, as the calcination temperature increased (comparing 400 °C and 500 °C), the degree of agglomeration of TiO_2_ decreased. The F-doped TiO_2_ particles prepared by firing at 500 °C were still uniform; the degree of agglomeration between the particles was low, and the nanoparticles were relatively uniform. However, agglomeration of undoped TiO_2_ prepared under the same conditions was aggravated, indicating that F-doping can effectively inhibit the agglomeration of TiO_2_ particles. In addition, after calcination at 500 °C, the F-doped TiO_2_ had smaller nanoparticles and exhibited good dispersibility. A better dispersibility can promote the activity of the photocatalyst.

### 3.2. Material Morphology and Elemental Signature

#### 3.2.1. F-Doped TiO_2_

The surface morphologies of TiO_2_ prepared at different calcination temperatures and TiO_2_ modified by doping with F are clearly shown on the SEM micrographs. In this experiment, a higher calcination temperature of 500 °C was used in order to reduce agglomeration and prepare more uniform and smaller photocatalyst nanoparticles. In addition to using SEM to understand the sample surface and its composition, in order to further study the morphology of the nanoparticles, a stronger electron beam was projected onto the TiO_2_ sample. Transmission electron microscopy (TEM) provides information about the internal structure of a sample, such as crystal structure, morphology, and stress state.

In addition to the special bamboo-like shape of the F-doped TiO_2_ prepared using EWP as the template, the nanoparticles were also very small, only 10–20 nm, and sometimes in the single digits at less than 10 nm. Compared with materials synthesized in other works, such as N, F-doped TiO_2_ nanomaterials at around 20 nm [25], and Zn-F-doped TiO_2_ [22,26], the F-doped TiO_2_ prepared using expired eggs in this experiment had a special bamboo-like shaped appearance and an unusual structure, and in particular the particles were also the smallest of those synthesized previously.

**Figure 3:**
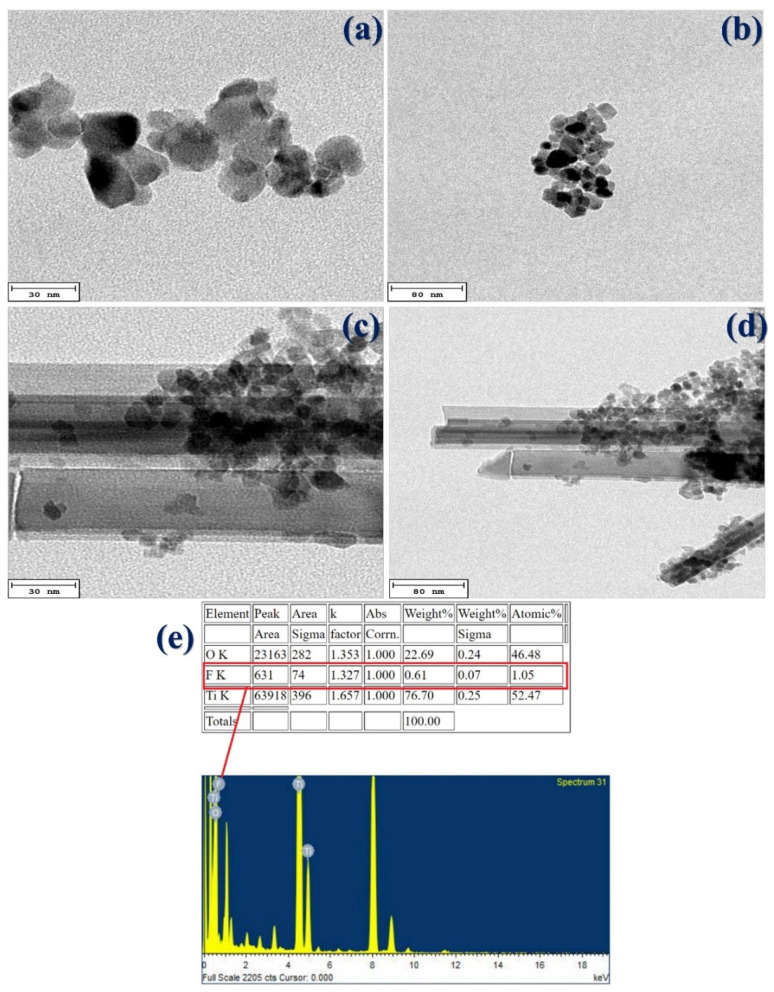
High resolution-transmission electron micrographs of bio-templated photocatalyst at 500 °C: (**a**) TiO_2_ (30 nm), (**b**) TiO_2_ (80 nm), (**c**) F-doped TiO_2_ (30 nm), (**d**) F-doped TiO_2_ (80 nm), and (**e**) High resolution-transmission electron micrographs energy dispersive X-ray spectrophotometer of F-doped TiO_2_.

The following Figure 3 shows TEM images for analysis and comparison: (a) using EWP as the template and (b) F-doped TiO_2_ nanoparticle structure morphology. TEM enabled visualization of the (a) undoped and (b) F-doped samples. It is obvious that there were large differences between the undoped and F-doped particles, whatever the preparation method. The size of the F-doped TiO_2_ nanoparticles was only 10~15 nm, smaller than the undoped TiO_2_ particles (about 20~30 nm on average).

#### 3.2.2. I-Doped TiO_2_

**Figure 4:**
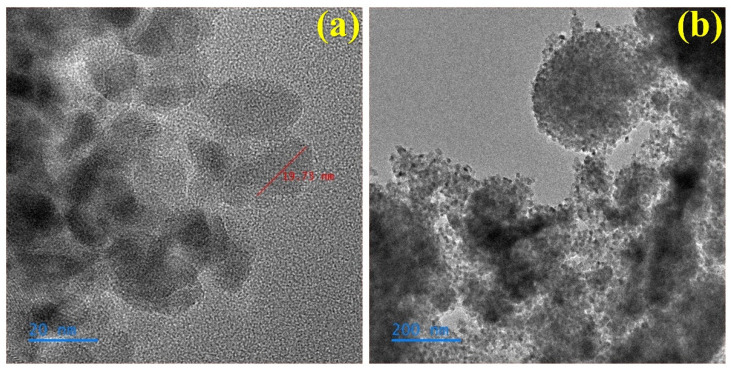
High resolution-transmission electron micrographs of bio-templated I-doped TiO_2_ photocatalyst at 500 °C: (**a**) 20 nm, (**b**) 200 nm.

In this study, in addition to using EWP as the template and NaF as an additive chemical for doping, NaI was also applied as a chemical agent to prepare I-doped TiO_2_. Analysis of the I-doped TiO_2_ showed in the UV-VIS-NIR test that there was no obvious absorption in the visible light region. However, the TiO_2_ nanoparticles prepared by doping with I were also relatively small in Figure 4. According to analysis of the results of TEM, the average size of this type of photocatalyst nanoparticle was 15–20 nm. It can be speculated that the smaller the catalyst particle, relatively, the larger the specific surface area, and this will play a greater role in the reaction efficiency of the photocatalyst.

In this work, HR-TEM-EDXS confirmed the presence of F-doped TiO_2_ composite material (Figure 3e). Analysis of the prepared F-doped TiO_2_ confirmed the existence of F atoms. F accounts for about 1.05% in photocatalyst products. In the case of F-doped TiO_2_ nanoparticles, an additional bamboo-like shape was exhibited in the HRTEM images, which could be responsible to reduce the size and increase the atomic percentage of synthesized materials. Therefore, it was confirmed that the elemental composition of the composite material F-doped TiO_2_ contained F atom in addition to Ti and O elements.

### 3.3. Electronic Interaction of Synthesized Material

**Figure 5:**
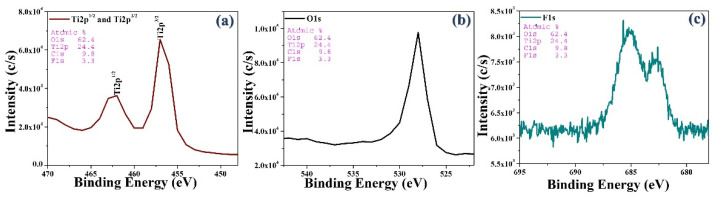
X-ray photoelectron spectra of bio-templated F-doped TiO_2_ photocatalyst at 500 °C (**a**) Ti2p^1/2 or, 3/2^, (**b**) O1s, and (**c**) F1s.

Using X-ray photoelectron spectroscopy (XPS), the surface composition and electronic interactions of synthetic materials, and relevant information about the bonding between two elements, can be obtained (Figure 5).

**Figure 6:**
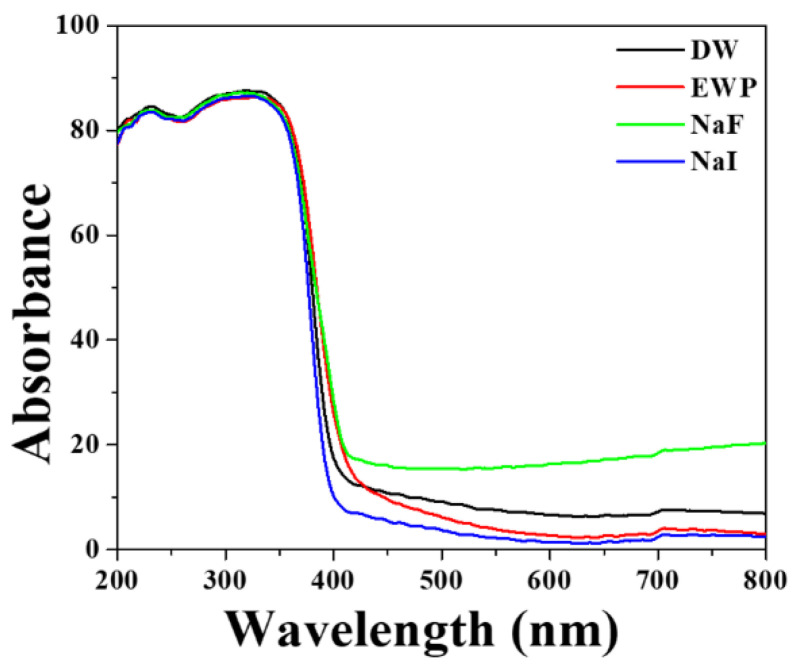
Ultraviolet-visible-near-infrared spectra of deionized water (DW), bio-templated TiO_2_ photocatalyst (EWP), and bio-templated F/I-doped TiO_2_ photocatalyst at 500 °C.

Using the best sample, the doping state of F and its light response wavelength were further analyzed. In order to understand and analyze the chemical state of the doped element F in the TiO_2_ lattice, in energy spectrum peak testing of F-doped TiO_2_, the sample with the highest photocatalytic degradation efficiency was employed. Figure 6 shows the XPS energy spectrum of F 1s of F-doped TiO_2_ prepared at a calcination temperature of 500 °C. According to data provided by the National Bureau of Standards and Technology of the United States and the results of analysis of related documents [27,28,29,30], it can be inferred that in the figure below, the peak at 681.5 eV shows the formation of F replacing O in the TiO_2_ lattice, the binding energy of the Ti-O-F chemical bond F 1s. In addition, the binding energy peak at about 685 eV is attributed to the F 1s binding energy of Ti-O-F formed when F interstitially enters the Ti-O lattice. This is consistent with the results reported in the literature [31,32].

### 3.4. Energy Charge Transfer and Optical Properties of the F-Doped TiO_2_ Synthesized Material

Figure 6 shows the UV-VIS-NIR absorption spectra of F/I-doped TiO_2_ and undoped TiO_2_ blanks prepared at a calcination temperature of 500 °C. It can be seen that the absorption edge of TiO_2_ after F doping exhibits an obvious red-shift, and the light response wavelength is extended to the visible light region. The absorption edge and widening of the forbidden band of TiO_2_ can be obtained by finding the wavelength and photon energy of the point corresponding to the maximum value of |dA/dλ| [33] (where A is the absorption rate and λ is the wavelength). It was calculated that the absorption edge of the F-doped TiO_2_ was red-shifted from 385 nm (before doping) to 428 nm. Obviously, the band gap was decreased from 3.2 eV (before doping) to 3.04 eV. The main reason for this should be that the 2p orbital energy of F doped in TiO_2_ is close to the 2p orbital energy of O in TiO_2_, which is prone to hybridization; this causes the valence band to shift upwards, resulting in a corresponding decrease in the band gap. Similar results were reported with the finding results of Wang et al. [34]. In addition, if the doped F introduces deep-level defects into the TiO_2_ energy band, the acceptor energy level is close to the top of the valence band, and the donor energy level is close to the bottom of the conduction band, meaning that electrons can indirectly transition from the valence band to the conduction band. The energy of the absorbed photons is hence reduced.

### 3.5. Photocatalytic Degradation of Methylene Blue (MB)

**Figure 7:**
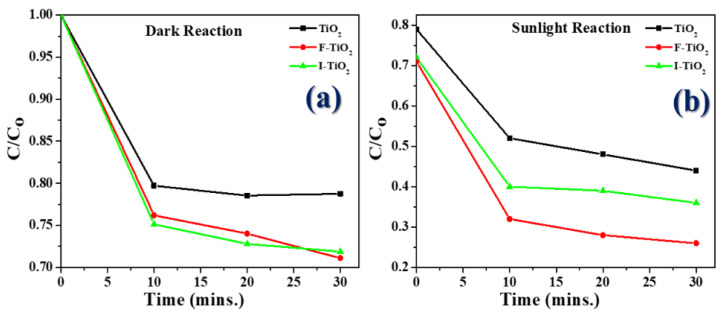
Photocatalytic degradation of MB using bio-templated TiO_2_, F-doped TiO_2_, and I-doped TiO_2_ photocatalyst. (**a**) dark room, (**b**) sunlight.

**Figure 8:**
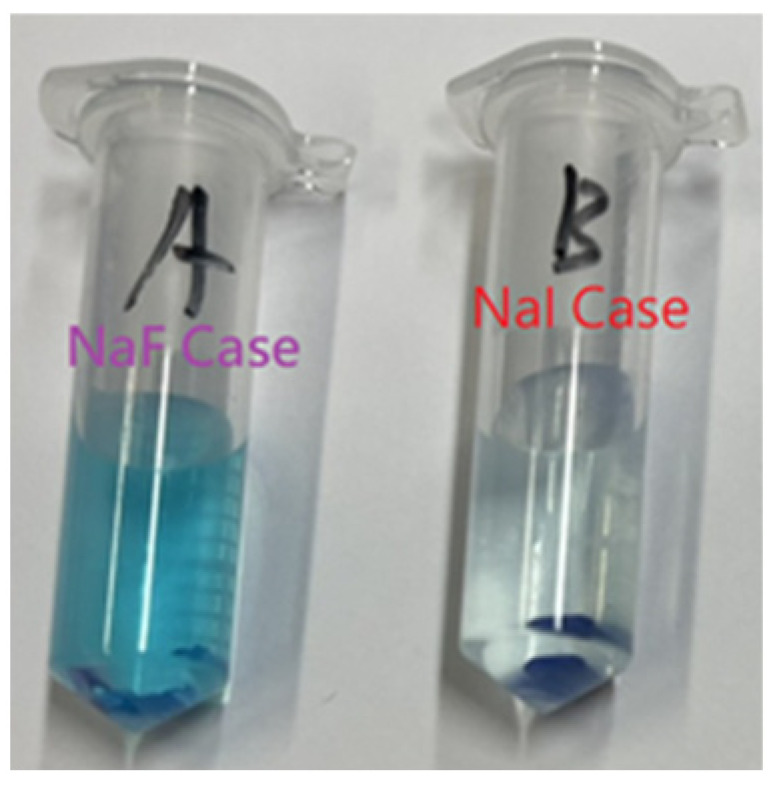
Dark blue solid residue was present at the bottom of the centrifuge tubes, (**A**) F-doped TiO_2_, and (**B**) I-doped TiO_2_ photocatalyst cases.

Regarding evaluation of the activity of the photocatalysts, in addition to using UV-VIS-NIR to compare the characteristic absorptions, methylene blue (MB) was also employed in this study in photocatalytic experiments in order to further understand the photocatalytic effects of the various TiO_2_ samples (Figure 7), where the observed color of doped sample was indicated in Figure 8.

The F-doped TiO_2_ photocatalytic material showed a high photocatalytic response not only under examination by UV-Vis, but also in the MB reaction test with a simulated sunlight source. These results agreed with those of another study, which showed F-doped TiO_2_ particles synthesized by the sol–gel method to enhance the absorption of visible light [35].

(a) In the first stage, the dark room test (Figure 7a), the catalyst particles of F-doped TiO_2_ and I-doped TiO_2_ (I-doped TiO_2_ more strongly) absorbed the blue color of MB; the value of C/Co dropped from 1 to 0.75, and the bottom of the centrifuge tubes was obviously dark blue, which was speculated to represent physical adsorption. Please refer to the image included (Figure 8); as can be seen, a dark blue solid residue was present at the bottom of the centrifuge tubes. It was thought that the residual TiO_2_ powder absorbed MB.
(b) In the second stage with simulated sunlight, after the reaction in the dark room was completed, various TiO_2_ samples were then irradiated with simulated sunlight. From the data shown in the figure (Figure 7b), it was apparent that the degradation rate of F-doped TiO_2_ was the fastest of all the samples, at up to 73%, followed by I-doped TiO_2_, and the degradation rate of TiO_2_ was the lowest at 44%. As simulated sunlight also contains a small part of UV light, TiO_2_ will also degrade in the MB aqueous solution test. The comparative removal percentage (%) of methylene blue dye has been shown in Table 1.

## 4. Reaction Mechanism

From the theory perspective of chemical MO (molecular orbital) and activation energy, the formation of O-F (single bond) could be easier than that of O=O (double bond), and the relative activation energy is lower. F-(fluorine atom with one electron) has an exceedingly stronger activity, and could be easily combined with O and become F-O bond (covalent molecular). In contrast, the binding energy of O=O is not easily breakable due to higher activation energy of O=O. Therefore, the O vacancy in the TiO_2_ lattice is easily replaced by F, and its activation energy is relatively small.

On the other hand, the hidden meaning is that electrons are more easily excited in this case. Relatively, the band gap between the CB and VB is narrowed. Therefore, we speculate that the F-doped TiO_2_ lattice can easily convert the photon energy (hυ) to chemical energy in the visible-light range due to narrow band gap of F-doped TiO_2_ produced in this study. However, the electrons can be easily excited on the surface of F-TiO_2_ photocatalyst.

**Figure 9:**
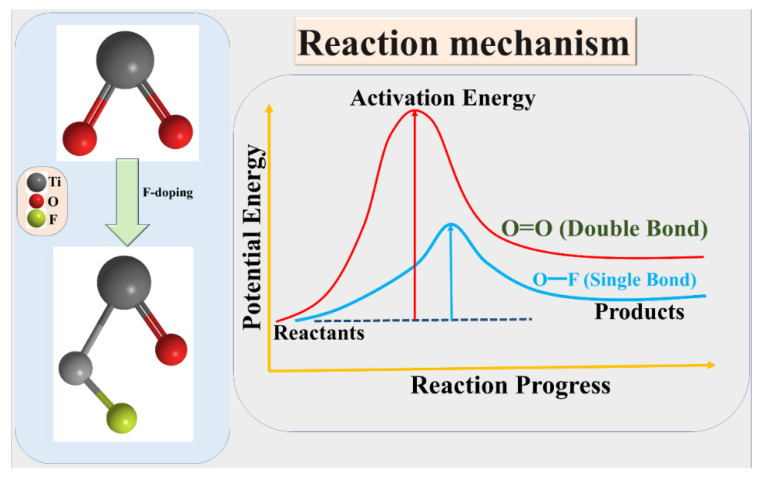
(**Left**) Doping of F into the molecular structure of TiO_2_, and (**Right**) the activation energies of the related single or double bonds.

The doping of F into the molecular structure of TiO_2_, and the activation energies of the related single or double bonds, are shown in Figure 9.

## 5. Conclusions

In current industrial applications, the main methods for synthesizing nanometer titanium dioxide powder are liquid-phase and gas-phase methods. Because traditional methods either are unable to be used to prepare nano-sized titanium dioxide, or the process is very difficult, a simpler process was designed in this study. Expired egg whites were employed as the template to prepare nano-TiO_2_ photocatalysts by the sol–gel method; in addition, NaF/NaI were employed in certain proportions for doping of the TiO_2_. The sol–gel method can be used to prepare single-component or multi-component molecular-level nano-catalysts of high purity, uniform particle size distribution, and high chemical activity at low temperature. Through UV/Vis and TEM, EDS, and XPS analyses, it was found that the prepared F-doped TiO_2_ powder had an absorption reaction under visible light; the MB reaction test confirmed this result.

F-doped TiO_2_ was prepared in this study. The F atom has one more electron than oxygen and replacing O_2_^−^ with F- in the lattice leaves an unpaired electron, as F can only accept one electron to obtain its closed shell structure. F-doped TiO_2_ has catalytic activity under visible light; in this study UV/Vis, TEM, and XPS analyses proved that the photocatalytic activity of the prepared F-doped TiO_2_ was better than that of P-25. However, the band gap was also observed as low as 3.04 eV. It is worthy of note that our process used expired eggs as the substrate, which is very in line with the issues of environmental protection and reuse. What is more, the F-doped TiO_2_ catalyst prepared in this study will be able to replace the commercial P-25 material, which is a topic worthy of development and research.

According to TEM, the particles of F-doped TiO_2_ were observed only 8.5 nm, which is smaller than the commercial P-25 particles; this means that the specific surface area of the F-doped TiO_2_ catalyst prepared has been increased. In addition, in terms of composition, approximately 1% (atomic) F was found to be present via XPS. In case of MB reaction test, it was observed that our product had approximately more than two times photocatalytic degradation percentage than that of P-25.

## Figures and Tables

**Table 1:** The comparative study of doped TiO_2_ nanoparticles.

Doped Nanoparticles	Contaminant Name	Light Source	Removal (%)	References
Pd–TiO_2_	Methylene blue and methyl orange	Visible light; 120 min	Methylene blue (99.4%) and methyl orange (92.6%)	[1]
V–TiO_2_	Methylene blue	Visible light; 300 min	15–30%	[2]
Carbon-doped TiO_2_	Methylene blue	Solar light	62.95%	[3]
Zirconium and silver co-doped TiO_2_	Methylene blue	Visible light	95%	[4]
F-doped TiO_2_	Methylene blue	Visible light	Discolored H^+^ (41.5%) and OH (46.5%)	[5]
F-doped TiO_2_ prepared by expired egg white	Methylene blue	LED light	73%	This study

## Data Availability

The data presented in this study are available on request from the corresponding author.
